# The First Survival Score for Patients Aged ≥80 Years Irradiated for Brain Metastases

**DOI:** 10.3390/biology11101434

**Published:** 2022-09-30

**Authors:** Dirk Rades, Cansu Delikanli, Steven E. Schild, Charlotte Kristiansen, Søren Tvilsted, Stefan Janssen

**Affiliations:** 1 Department of Radiation Oncology, University of Lubeck, 23562 Lubeck, Germany; 2 Department of Radiation Oncology, Mayo Clinic, Scottsdale, AZ 85259, USA; 3 Department of Oncology, Vejle Hospital, University Hospital of Southern Denmark, 7100 Vejle, Denmark; 4 Research Department, Zealand University Hospital, 4600 Køge, Denmark; 5 Medical Practice for Radiotherapy and Radiation Oncology, 30161 Hannover, Germany

**Keywords:** brain metastases, very elderly, radiotherapy, survival, prognostic tool

## Abstract

**Simple Summary:**

Decisions regarding personalized treatment for brain metastases should consider the patient’s lifespan, which can be estimated with survival scores. Since very elderly patients (≥80 years) are different from other patients, e.g., due to a higher comorbidity index and decreased organ functions, these patients likely benefit from specific scores. A survival score was generated in 94 patients aged ≥ 80 years who were irradiated (whole-brain radiotherapy) for metastases of the brain. The score achieved high accuracy with respect to the prediction of death up to 6 months and survival for ≥1 and ≥2 months following treatment. When compared to an existing tool, the new score was more precise regarding death ≤ 1 month following radiotherapy and survival (all time points). Therefore, the new score appears preferable.

**Abstract:**

Survival scores facilitate personalized cancer treatment. Due to demographic changes, very elderly patients are more prevalent than in the past. A score was developed in 94 patients aged ≥ 80 years undergoing whole-brain radiotherapy for brain metastases. Dose fractionation, treatment period, age, sex, performance score (ECOG-PS), tumor type, count of lesions, metastases outside the brain, and interval tumor diagnosis to radiotherapy were retrospectively evaluated. Independent predictors of survival were used for the score. Based on individual scoring points obtained from 3-month survival rates, prognostic groups were designed. Additionally, the score was compared to an existing tool developed in patients ≥ 65 years. ECOG-PS, count of lesions, and extra-cranial metastases were independent prognostic factors. Three groups were created (7, 10, and 13–16 points) with 3-month survival of 6%, 25%, and 67% (*p* < 0.001), respectively. Positive predictive values (PPVs) regarding death ≤ 3 and survival ≥ 3 months were 94% and 67% (new score) vs. 96% and 48% (existing tool), respectively. PPVs for survival ≥1 and ≥2 months were 88% and 79% vs. 63% and 58%, respectively. Both tools were accurate in predicting death ≤2, ≤3, and ≤6 months. The new score was more precise regarding death ≤1 month and survival (all time periods) and appeared preferable. However, it still needs to be validated.

## 1. Introduction

A substantial number of adult cancer patients experience brain metastases [1]. Many of them receive radiotherapy, either alone or following neurosurgical resection. Several types of radiotherapy are available, including whole-brain radiotherapy (WBRT) and local irradiation, namely, single-fraction radiosurgery (SRS) and fractionated stereotactic radiotherapy (FSRT) [1]. In most studies, SRS and FSRT were limited to 1–4 cerebral lesions. Although studies from Japan showed that SRS also appeared safe for 5–10 lesions, the majority of these patients receive WBRT alone, particularly if they are older or frail, or if uncontrolled extra-cranial metastases exist [1,2]. Moreover, local therapies are generally limited to lesions with a diameter of ≤4 cm [1]. In the case of a high risk of new brain metastases outside of the irradiated sites, local therapies and WBRT may be combined to improve intracerebral control [3]. However, one should be aware that adding WBRT is associated with more pronounced neurocognitive decline [4]. 

For WBRT alone, several dose-fractionation regimens are available [1]. The most appropriate individual regimen depends on several factors including the survival prognosis. Generally, patients with a very limited life expectancy should receive a less time-consuming regimen to ensure that they spend as little time as possible of their remaining lifespan receiving WBRT. Patients with very limited survival times may not benefit from WBRT at all and be treated with best supportive care (BSC) including corticosteroids [5]. In contrast, for patients with more favorable prognoses, longer-course regimens with higher total doses and lower doses per fraction are more appropriate [1]. Higher total doses may lead to longer-lasting intracerebral control, and lower doses per fraction are associated with a decreased risk of late sequelae including neurocognitive decline [6,7,8,9]. Since the risk of late effects increases with time, this endpoint is more important for longer-term survivors. These considerations illustrate that it is important for the treating physicians to estimate individual survival prognoses most precisely prior to the selection of an individual therapy. This estimation will be considerably facilitated with scoring instruments. Several instruments are available for the radiation treatment of brain metastases including a survival score for patients aged ≥ 65 years [10]. Such a score is reasonable, since patients of this age group are different from younger patients, e.g., due to higher degrees of comorbidity including cardiovascular disease and decreased organ functions. This holds particularly true for the group of very elderly patients. Several authors defined very elderly as ≥80 years, and some of them compared this age group to patients aged 70 to 79 years [11,12,13,14,15,16,17]. Due to the demographic change, the group of very elderly patients is growing and will come increasingly into focus [18]. To provide a very good personalized treatment for these patients, the development of specific prognostic tools for this group appears reasonable. An instrument has now been created in cancer patients ≥ 80 years assigned to radiotherapy of brain metastases. To reduce the risk of a selection bias due to radiotherapy type, only WBRT alone was allowed for eligibility. The novel score was primarily designed to predict 3-month survival probability, but should also be usable to estimate the survival at 1, 2, and 6 months. In addition, the score was compared for accuracy with a previous tool developed for patients aged ≥ 65 years.

## 2. Patients and Methods

Ninety-four patients aged 80 years or older, who received WBRT without neurosurgical resection or radiosurgery at two German radiotherapy departments between 2000 and 2021, were retrospectively evaluated. The dose-fractionation regimen of radiotherapy and seven characteristics were evaluated for associations with survival following radiotherapy. Performance status was represented by the Eastern Cooperative Oncology Group performance score (ECOG-PS), which was obtained from the patient records. In addition, since systemic treatments have changed over time, which might have had an impact on the survival outcomes, patients treated between 2000 and 2012 (*n* = 48) were compared to those treated between 2013 and 2021 (*n* = 46). The distribution of the dose-fractionation regimens and the other characteristics are summarized in Table 1.

Univariate analyses were performed using the Kaplan–Meier method and the log-rank test. The characteristics that achieved significance (*p* < 0.05) were analyzed for independence using a Cox proportional hazards model. *p*-values < 0.05 indicated significance. The characteristics identified as independent predictors of survival were incorporated in the scoring tool; the 3-month survival rates given in percent were divided by 10. The resulting points were summed for each individual to obtain patient scores. Higher scores represented better survival outcomes. Depending on the 3-month survival rates of the patient scores, three groups were designed.

In addition, the new score was compared to an existing tool (Evers-Score), previously developed for elderly patients aged 65 years or older [10]. Comparisons for diagnostic accuracy were performed using the positive predictive values (PPVs) to correctly identify individuals dying within 1, 2, 3, and 6 months, respectively (worst prognostic groups), and individuals surviving for 1, 2, 3, and 6 months, respectively (best prognostic groups).

The PPVs for the prediction of death were calculated using the following equation:

PPV = [patients dying within n* mos./(patients dying within n* mos. + patients not dying within n* mos.)] × 100 (1)

The PPVs for the prediction of survival were calculated using the following equation:

PPV = [patients surviving for *n* mos./(patients surviving for n* mos. + patients not surviving for n* mos.)] × 100 (2)

[mos. = months; *n = 1, 2, 3 or 6 months, respectively].

## 3. Results

**Figure 1:**
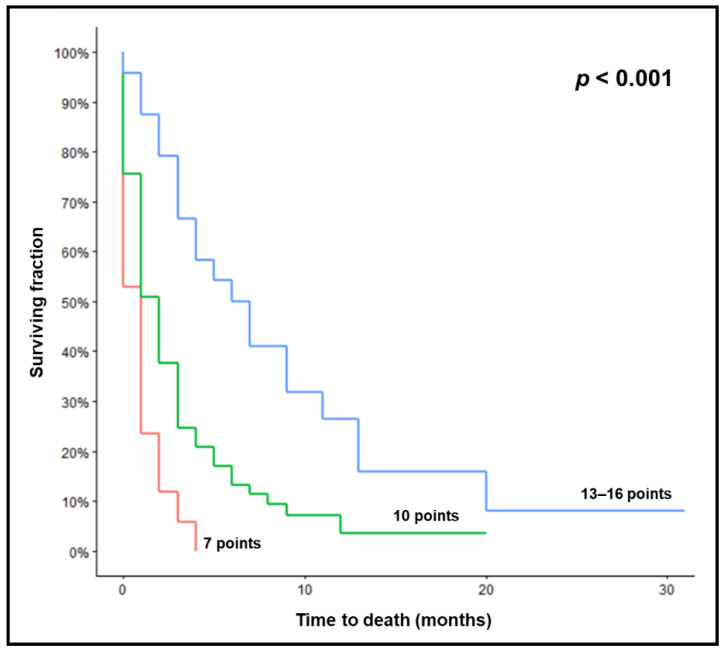
Kaplan–Meier curves for survival of the three prognostic groups (7, 10, and 13–16 points). *p*-value was calculated using the log-rank test.

The median survival was 2.0 months in the whole series. On univariate analyses, a better ECOG performance score (*p* = 0.003), lower number of brain metastases (*p* = 0.014), and no metastases outside the brain (*p* = 0.034) were significantly associated with improved survival (Table 2). Since survival rates at 3 months were similar for ECOG-PS 0–1 and ECOG-PS 2 and the score was based on the 3-month survival rate, these were combined, although ECOG 0–1 achieved better outcomes at 6 months (Table 2). The three significant characteristics, i.e., ECOG-PS (0–2 vs. 3; hazard ratio 1.52; 95% confidence interval 1.18–1.95; *p* = 0.001), number of brain metastases (1 vs. 2–3 vs. ≥4; 1.32; 1.10–1.58; *p* = 0.003) and extra-cranial metastases (no vs. yes; 2.47; 1.42–4.29; *p* = 0.001), were also significant in the multivariate analysis and incorporated into the survival score. The scoring points are given in Table 3. After the addition of the points for individual patients, the scores were 7 (*n* = 17), 10 (*n* = 53), 13 (*n* = 21), and 16 (*n* = 3) points, respectively. The corresponding 3-month survival rates were 6%, 25%, 62%, and 100% (*p* < 0.001). Since the number of patients with 16 points was small, 13 and 16 points were combined and three groups were created: 7 points (*n* = 17), 10 points (*n* = 53), and 13–16 points (*n* = 24). The median survival times were 1.0, 2.0, and 6.5 months, respectively (Figure 1, *p* < 0.001). The survival rates at 1, 2, 3, and 6 months of these groups are shown in Table 4. When performing intergroup comparisons for 3-month survival (Fisher’s exact test), the difference between the 10-point and the 13–16-point groups was significant (*p* < 0.001), and the comparison between the 7-point and the 10-point groups showed a trend (*p* = 0.16). In the 10-point group, seven patients survived >6 months. Six of these patients were younger than 85 years, five patients had less than four lesions, and five patients an ECOG-PS of 0–1.

The new tool was compared to the Evers-Score, which originally included four prognostic groups (3–6, 7–9, 10–12, and 13 points, respectively) [10]. Since only two patients of the present cohort achieved 13 points on the Evers-Score, the two best prognostic scores (10–12 and 13 points) were combined to one group. This procedure facilitated the comparability of both tools. The PPVs for both correct prediction of death within 1, 2, 3, and 6 months and the survival of at least 1, 2, 3, and 6 months are summarized in Table 5. The Evers-Score differentiated between the involvement of 0 (4 scoring points), 1 (3 points), or ≥2 (1 point) extra-cranial organs, and not between the absence or presence of extra-cranial metastases (present score). Since, in the present study, the count of involved extracranial organs was not available for 42 patients (45%), patients with extra-cranial metastases were assigned 2 points when calculating the Evers-Score. Given this limitation, both scores performed almost equally with respect to the prediction of death within 2, 3, and 6 months. The new score appeared more accurate regarding death within 1 month and survival for 1, 2, 3, and 6 months. Thus, it appears preferable for patients of 80 years or older. However, since the survival was poor in the entire series, the new instrument achieved high PPVs to predict survival ≥1 month (88%) and ≥2 months (79%), but not ≥3 months (67%) and ≥6 months (50%).

## 4. Discussion

The number of very elderly cancer patients (≥80 years) is constantly growing. Patients of this age group often have significant comorbidities. Moreover, liver and kidney function and bone marrow reserve are often worse than in younger patients. Therefore, very elderly patients may be less resistant to aggressive cancer therapies and less compliant. For example, in a study of breast cancer patients, women ≥80 years were significantly less likely to receive the recommended adjuvant radiotherapy than patients aged 70–79 years [19]. To avoid over- or undertreatment and provide optimal treatment for very elderly patients, they should be considered a separate group. This also applies to palliative situations such as the occurrence of brain metastases.

Several improvements regarding the treatment of brain metastases have been achieved during the last one or two decades, including high-precision radiotherapy techniques and novel targeted systemic therapies [1,20]. Another important and comparably novel approach is the design of personalized treatment regimens. This concept particularly considers an individual patient’s needs, preferences, and social situation. Moreover, the patient’s survival prognosis is important. In case of very limited prognosis, the patients should receive a treatment that is both short and little burdensome, or BSC including dexamethasone and analgesics [5]. The more favorable the survival prognosis, the more important local control, survival, and longer-term treatment-related toxicity become. Therefore, it is relevant to be able to estimate an individual patient’s remaining lifespan. For patients with brain metastases, several survival scores are available including a tool for elderly patients aged ≥65 years [10,21,22,23]. Until now, no survival score was developed specifically for very elderly patients with brain metastases. The present study was performed to close this gap. In 94 patients aged ≥80 years who underwent WBRT alone, a survival score including three prognostic groups was created.

Patients of the 7-point group had very limited survival times. Only 12% and 6% survived ≥2 and ≥3 months, respectively, and all patients died within 4 months. Therefore, these patients should receive BSC alone, including dexamethasone and analgesics. In the Quality of Life after Treatment for Brain Metastases (QUARTZ) trial, performed in 538 patients with brain metastases from lung cancer, WBRT when added to BSC plus dexamethasone provided very little clinical benefit [5]. The quality-adjusted life-years were only 46.4 and 41.7 days, respectively. There were no significant differences regarding serious adverse events, survival, overall quality of life, and administration of dexamethasone.

Patients of the 10-point group also had unfavorable prognoses with a median survival of 2.0 months, and 3- and 6-month survival rates of 25% and 13%, respectively. The median survival time of 2.0 months is similar to those times observed in the QUARTZ trial (9.2 weeks in the WBRT group and 8.5 weeks in the BSC-alone group, respectively) [5]. Thus, many patients of the 10-point group appear to be suitable candidates for BSC including dexamethasone and analgesics. Selected patients may receive WBRT with 5 × 4 Gy. In a retrospective study of 442 patients treated with WBRT for >3 lesions, 5 × 4 Gy was no less effective than 10 × 3 Gy regarding 6-month survival (24% vs. 27%, *p* = 0.29) and intracerebral control (50% vs. 37%, *p* = 0.07) [24]. WBRT in addition to BSC should be considered particularly for patients younger than 85 years, patients with <4 cerebral lesions, and/or patients with an ECOG-PS of 0–1.

Patients of the 13–16-point group had the best survival, e.g., a 6-month survival rate of 50%. Moreover, 27% of these patients survived for ≥12 months. Therefore, these patients can benefit from longer-course radiotherapy such as 20 × 2 Gy. In a study from 1989, neurocognitive deficits following WBRT were observed, when doses per fraction of ≥3 Gy were used [6]. Shibamoto et al. recommended 20 × 2 Gy in their review article [7]. In 2019, Gaspar et al. considered 10 × 3 Gy the standard regimen and recommended regimens including higher doses per fraction (e.g., 5 × 4 Gy) only in cases of a poor performance status or very limited survival due to concerns regarding neurocognitive decline [8]. In contrast, a post hoc analysis of a phase 3 trial that compared the neurosurgical resection of a single lesion followed by SRS (*n* = 102) or WBRT (*n* = 92), the WBRT regimen (15 × 3.5 Gy or 10 × 3 Gy) showed no significant impact on neurocognitive function [25]. However, since these patients only had a single lesion and 20 × 2 Gy was not used, these results may not apply to patients not suitable for neurosurgery or SRS, but WBRT alone. Patients of the 13–16-point group with 1–4 lesions may also be considered for SRS or FSRT alone. Two retrospective studies suggested that SRS was effective and feasible in patients aged ≥ 80 years [16,17].

When following the recommendations made above, one has to be aware of the limitations of this study. Despite the fact that only patients receiving WBRT were included to reduce the risk of bias caused by the type of radiotherapy, such a risk remains due to the retrospective study character. Due to the smaller number of subjects in the age group ≥80 years compared to other age groups, the present score could not be validated. There might be a significant difference in survival between ECOG-PS 0–1 and ECOG-PS 2 in a larger cohort. Moreover, despite a notable survival difference of 19% at 3 months between the 7-point and the 10-point groups, this difference did not achieve significance. However, despite these limitations, the new score, which is the first tool specifically designed for very elderly patients irradiated for cerebral metastases, appeared more accurate than a previous score developed in elderly patients aged ≥ 65 years [10].

## 5. Conclusions

The first survival score specifically designed for patients aged ≥ 80 years with brain metastases is presented. Given its limitations, the new instrument was very accurate in predicting death ≤1, ≤2, ≤3, and ≤6 months following radiotherapy and survival for ≥1 and ≥2 months. Since the survival was comparably poor in the entire cohort, the PPVs regarding the prediction of survival for ≥3 and ≥6 months were lower. When compared to a previous tool created for patients aged ≥65 years, the new score was more accurate in predicting death ≤1 month and survival within all four periods of time. Thus, the new instrument was considered preferable for patients aged ≥80 years. It can help physicians to design the optimal individual therapy for patients of this age group. Since the new instrument was specifically designed for patients ≥ 80 years of age, it should not be used for younger patients, particularly not for patients < 65 years. Moreover, one has to be aware that the score still requires validation, ideally in a larger prospective cohort, which is important for its introduction into clinical routine.

## Figures and Tables

**Table 1:** Characteristics evaluated for survival.

Characteristic	Number of Subjects	Proportion (%)
Age		
80–84 years	68	72
85–90 years	26	28
Gender		
Female	39	41
Male	55	59
ECOG performance score		
0–1	30	32
2	36	38
3	28	30
Tumor type		
Breast cancer	10	11
Non-small cell lung cancer	42	45
Small-cell lung cancer	9	10
Less radiosensitive tumors	6	6
Cancer of unknown primary	6	6
Gastrointestinal cancers	9	10
Other malignancies	12	13
Number of brain metastases		
1	18	19
2–3	24	26
≥4	52	55
Extra-cranial metastases		
No	20	21
Yes	74	79
Interval tumor diagnosis to WBRT		
≤12 months	65	69
>12 months	29	31
Treatment period (years)		
2000–2012	48	51
2013–2021	46	49
WBRT regimen		
20 Gy in 5 fractions	12	13
30 Gy in 10 fractions	40	43
>30 Gy in 11–20 fractions	42	45

ECOG: Eastern Cooperative Oncology Group; WBRT: whole-brain radiotherapy.

**Table 2:** Survival rates at 1, 2, 3, and 6 months following radiotherapy related to patient characteristics, treatment period, and dose-fractionation regimen.

Characteristic	Survival Rates (%)				*p*-Value
	At 1 Month	At 2 Months	At 3 Months	At 6 Months	
Age					0.54
80–84 years	50	44	35	22	
85–90 years	69	42	23	15	
Gender					0.83
Female	51	46	31	23	
Male	58	42	33	18	
ECOG performance score					0.003
0–1	57	50	40	33	
2	61	53	42	25	
3	46	25	11	0	
ECOG performance score					<0.001
0–2	59	52	41	29	
3	46	25	11	0	
Tumor type					0.73
Breast cancer	50	40	40	30	
Non-small cell lung cancer	55	45	29	24	
Small-cell lung cancer	44	44	22	11	
Less radiosensitive tumors	67	50	33	17	
Cancer of unknown primary	100	83	67	17	
Gastrointestinal cancers	44	22	22	11	
Other malignancies	50	33	33	17	
Number of brain metastases					0.014
1	94	78	56	44	
2–3	50	38	29	25	
≥4	44	35	25	10	
Extra-cranial metastases					0.034
No	80	65	55	30	
Yes	49	38	26	18	
Interval from tumor diagnosis to WBRT					0.11
≤12 months	54	42	28	14	
>12 months	59	48	41	34	
Treatment period (years)					0.29
2000–2012	63	48	35	21	
2013–2021	48	39	28	20	
Dose-fractionation regimen					0.66
20 Gy in 5 fractions	83	58	50	8	
30 Gy in 10 fractions	55	40	30	20	
>30 Gy in 11–20 fractions	48	43	29	24	

ECOG: Eastern Cooperative Oncology Group; WBRT: whole-brain radiotherapy.

**Table 3:** Survival rates at 3 months and scoring points.

Characteristic	Survival Rate at 3 Months (%)	Scoring Points
ECOG performance score		
0–2	41	4
3	11	1
Number of brain metastases		
1	56	6
2–3	29	3
≥4	25	3
Extra-cranial metastases		
No	55	6
Yes	26	3

ECOG: Eastern Cooperative Oncology Group.

**Table 4:** Three prognostic groups and corresponding survival rates at 1, 2, 3, and 6 months.

	Survival Rates (%)				*p*-Value
	At 1 Month	At 2 Months	At 3 Months	At 6 Months	
Prognostic group					**<0.001**
7 points	24	12	6	0	
10 points	51	38	25	13	
13–16 points	88	79	67	50	

Bold *p*-value is significant.

**Table 5:** Comparison of the new instrument and the previous Evers-Score [10] with respect to accuracy to predict death within 1, 2, 3, and 6 months (worst prognostic groups) and survival for at least 1, 2, 3, and 6 months (best prognostic groups).

Endpoint	PPVs of the New Score	PPVs of the Evers-Score
Death		
within 1 month	76%	56%
within 2 months	88%	80%
within 3 months	94%	96%
within 6 months	100%	100%
Survival		
for at least 1 month	88%	63%
for at least 2 months	79%	58%
for at least 3 months	67%	48%
for at least 6 months	50%	35%

PPVs: positive predictive values.

## Data Availability

The data analyzed for this paper cannot be shared due to data protection regulations.
